# Extranodal involvement in lymphoma – A Pictorial Essay and Retrospective Analysis of 281 PET/CT studies

**Published:** 2014

**Authors:** Jayanta Das, Soumendranath Ray, Saugata Sen, Mammen Chandy

**Affiliations:** 1 Nuclear Medicine and PET/CT, Tata Medical Center, New Town, Kolkata, India; 2 Radiology Department, Tata Medical Center, New Town, Kolkata, India; 3 Haematology Department, Tata Medical Center, New Town, Kolkata, India

**Keywords:** Hodgkin’s disease, Lymphoma, PET/CT

## Abstract

**Objective(s):**

The aim of this study is to evaluate the role of PET-CT in identification of different patterns of extranodal involvement in Hodgkin’s disease (HD) and Non-Hodgkin’s Lymphoma (NHL) and to enlist the common sites of extranodal involvement in each histological type and compare our results with the existing literature.

**Methods:**

In this retrospective study of 281 cases of lymphomas of various histologies, we illustrate the spectrum of PET/CT features of extranodal lymphoma (ENL) of commonly involved organs and compare our result with the literature.

**Result:**

Extranodal appearance in lymphoma is strikingly varied. Diffuse large B cell lymphoma (DLBCL) is the commonest histological subtype and gastrointestinal tract is the commonest anatomical subsite in NHL. Skeletal system is the commonest site for involvement in HD.

**Conclusion:**

A broad spectrum of extranodal organs is involved in various subtype of lymphoma which can be depicted in PET-CT in the most appropriate manner. Familiarity with the pattern of involvement is essential for comprehensive management.

## Introduction

Commonly, lymphoma originates in lymph nodes. Infiltration of malignant lymphomatous cells in the organs other than lymph node is termed as extranodal lymphoma. Almost any organ in the body can be affected. The most frequently involved system is GI tract (Stomach being the commonest site) followed by Waldeyer’s ring (when tonsil is considered as an extranodal site), lung, liver, spleen, bone and and skin (1). Primary CNS lymphoma is also well documented. Origin of tumor from non lymph-nodal tissue is termed as primary extranodal lymphoma (ENL), whereas hematogenous spread of disease from lymph nodes to extranodal tissue is secondary extranodal lymphoma (2). Extranodal involvement is more common in Non Hodgkin’s Lymphoma (NHL) than Hodgkin’s disease (HD).

Incidence of extranodal disease is rising (3, 4). Various factors have been attributed to this changing trend of lymphoma namely HIV, increasing use of immunosuppressive therapy and indolent viral infection.

Extranodal disease is prognostically important in any lymphoma. HD is usually confined to the lymph nodes. Extralymphatic extension or involvement of spleen in a case of primary nodal disease upstages the disease in group III. National cancer database report on NHL shows patient with primary extranodal disease tend to present in lower stage than the primary nodal disease (5).

Accurate localization and staging of the disease are essential for deciding the treatment strategy (5-7). Conventional cross sectional imaging have various limitations in evaluation of lymphomas. PET with the ability to image the metabolically active tumour is being routinely used in evaluation of lymphoma in past two decades. FDG PET/CT is now the imaging modality of choice for staging and follow up of both Hodgkin’s disease as well as NHL (1)

In this article, we discuss involvement of various common and uncommon organs in extra-nodal lymphoma with particular attention to PET/CT findings. The pictorial essay covers a broad spectrum of extra-nodal disease including various organs of the body.

## Methods

A retrospective review was conducted in 281 patients with histologically proved lymphoma who underwent PET/CT in our institution between September 2011 and October 2012. All studies were done as per guideline of indication of PET/CT in lymphoma (8).

Whole body PET-CT imaging was performed from vertex of the skull to midthigh approximately 60 minutes after intravenous injection of 10-12 mCi of ^18^F-FDG using a BGO PET camera (GE, 64 slice DISCOVERY VCT). Children were injected proportionally lesser dose. Blood sugar level was estimated routinely before the study and it was ensured that it remains below 160 mg/dl. PET images were acquired at a rate of 2 minutes per bed position. Contrast enhanced CT was performed in all cases unless iodinated contrast was contraindicated.

Follow up data were available in most of the patients Images were interpreted in both subjective and objective methods. SUV values of 3 and more were considered positive for malignancy. SUV values greater than 2.5 but less than 3 were interpreted in correlation with clinical findings and CT scan appearances. Histopathological confirmation was not routinely performed in every lesion. Diagnosis was reached by consensus of physicians and that multiple organs involvement made other possibilities unlikely.

The images were interpreted and data analysed by a Nuclear Medicine physician, two Radiologists and two Haematologists.

## Results

Out of 281 patients, 185 (65.8%) were NHL and 96 (34.1%) were HD. Commonest histological subtype of HD group was mixed cellularity involving 23.9 % and nodular sclerosis in 20% of all HD cases. Twenty three patients were of any one of the classical subtype where detail histological information could not be obtained (patients from other institutions).

Among the NHL patients, commonest histological subtype was DLBCL comprising 51.3% of all NHL cases and comprises 33.8% of total study cohort followed by follicular subtype (14%).

In our study, 42.7% of patients (120 cases) were found to have extranodal involvement and 94 patients (78.3%) had NHL.

Primary extranodal involvement was found in 61 (21.7%) patients; of which 93.4 % had NHL Each of the other subtypes includes less than 5% of patients.

Secondary extra nodal involvement was found in 59 patients (20.9 % of all cohort) of which 37 (62.7%) had NHL.

DLBCL is the commonest histological subtype in both primary as well as secondary extranodal lymphoma group and comprised of 44.1% of total extranodal lymphoma. 57.3% of primary extranodal lymphoma and 30% of secondary extranodal lymphoma are of DLBCL subtype.

### Non Hodgkin Lymphoma (NHL)

**Figure 1:**
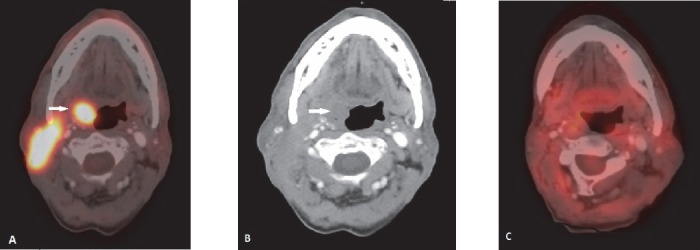
NHL involving tonsil: Asymmetric radio tracer uptake in right tonsil (arrow in A). CT scan shows fullness in right tonsilar fossa (arrow in B). Metabolically active enlarged right cervical lymph node is also seen. Post 3 cycle chemotherapy shows complete metabolic response (C)

**Figure 2:**
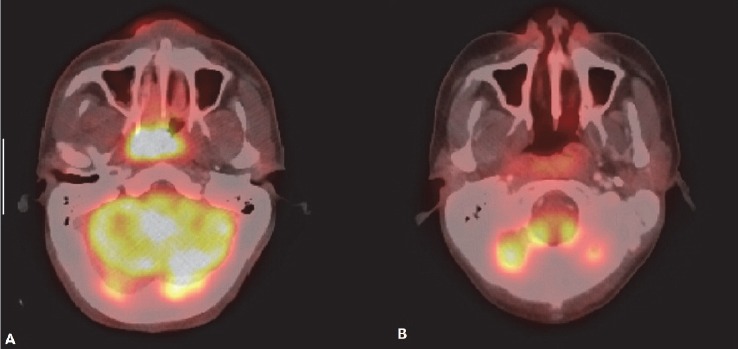
NHL of nasopharynx: (A) FDG avid soft tissue mass is seen in right posterolateral wall of the nasopharynx with obstruction of right posterior nasal choana. (B) Post chemotherapy scan for response assessment shows complete metabolic response

**Figure 3:**
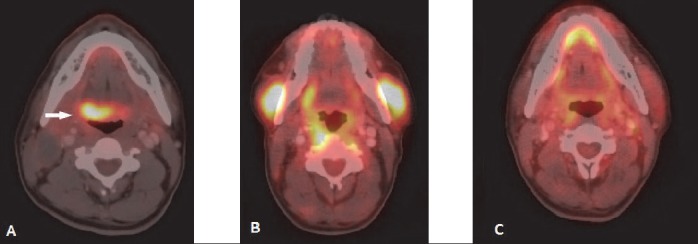
Lymphoma of tongue: (A) FDG avid plaque like lesion is seen in posterior tongue on right side (arrow). (B) Post 3 cycle chemotherapy shows partial metabolic response. Diffuse FDG uptake of both submandibular glands indicates chemotherapy induced sialadenitis. (C) Follow up scan few weeks after completion of 6 cycle of chemotherapy shows complete metabolic response. Both submandibular glands show normal appearance

**Figure 4:**
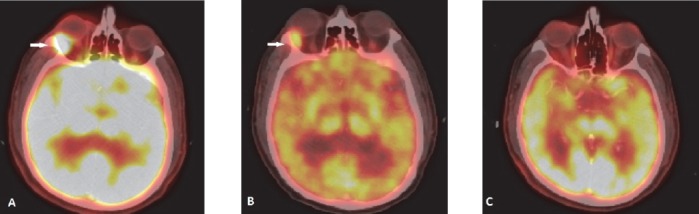
NHL of orbit: (A) FDG avid soft tissue lesion is seen in extraconal compartment of right eye not separable from lateral rectus muscle. (B) Post 3 cycles Bendamustine shows partial metabolic response. (C) Follow up scan after 6 cycles of chemotherapy shows no residual lesion

**Figure 5:**
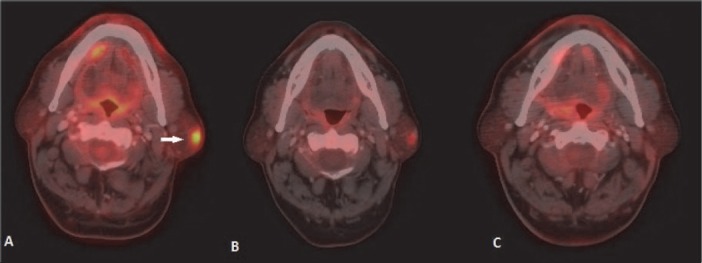
Lymphoma of left parotid gland: Same patient as in figure 4. (A) Relapsed follicular lymphoma shows FDG avid nodule in superficial lobe of the left parotid gland. Patient also had nodal disease including cervical nodes. (B) Post 3 cycles Bendamustine shows complete metabolic response. (C) Follow up scan after 6 cycles of chemotherapy shows no residual lesion in left parotid gland

Head and neck region was involved in 18 cases of NHL (19.1% of all extranodal NHL cases). However, Waldeyer’s ring was the commonest site in head and neck region, tonsil being the most common affected organ involved in 8.5% of all extranodal NHL patients (Figure 1). Nasopharynx was involved in 3.1% (Figure 2). Tongue in 2.1 % (Figure 3) and orbit in 3.1 % (Figure 4). Other sites of involvement in head and neck were sinonasal cavity, thyroid cartilage and parotid gland (Figure 5).

**Figure 6:**
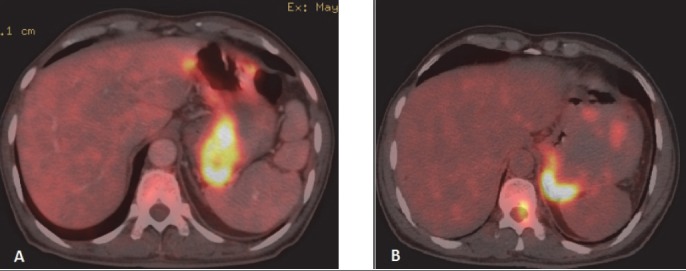
NHL of stomach. FDG avid plaque like thickening of wall of stomach is seen along the greater curvature of the stomach (A). Repeat scan during post therapy response assessment shows partial metabolic response (B)

**Figure 7:**
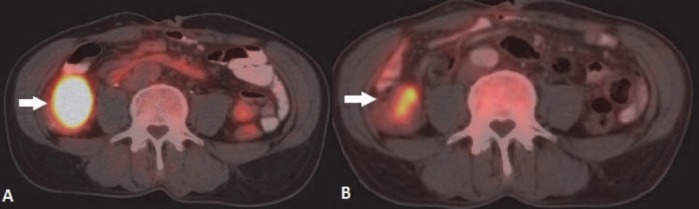
NHL of right colon. FDG avid circumferential mural thickening of caecum near the iliocaecal junction is seen (A). Post therapy scan (B) show partial response

The most frequently involved extranodal organ was gastrointestinal tract (GIT) with 14.8% of patients. Within the GIT, stomach was the commonest site 8.5% (Figure 6). Small intestine was involved in 4.2% with terminal ileum being the commonest site. Colon was involved in 2 patients (2.1%) (Figure 7).

**Figure 8:**
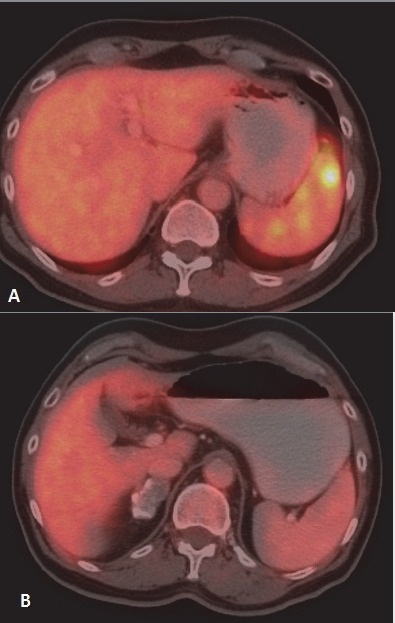
Same patient as in Fig 7. FDG avid focal lesion in spleen (A) shows complete metabolic as well as anatomical response in post treatment scans (B)

**Figure 9:**
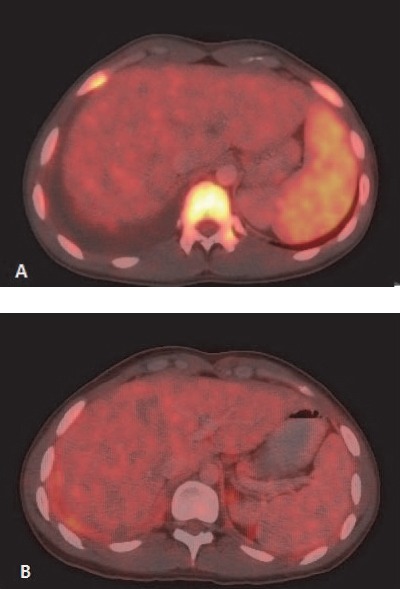
Diffuse involvement of spleen in a case of NHL is seen (A). Note greater FDG avidity of spleen as compared to liver. Post therapy scan shows complete metabolic response (B)

8.5% of all cases of extranodal NHL had hepatic involvement. Spleen was involved in 12.7% and focal involvement was more common (Figure 8), but diffuse involvement was also seen in few cases (Figure 9).

**Figure 10:**
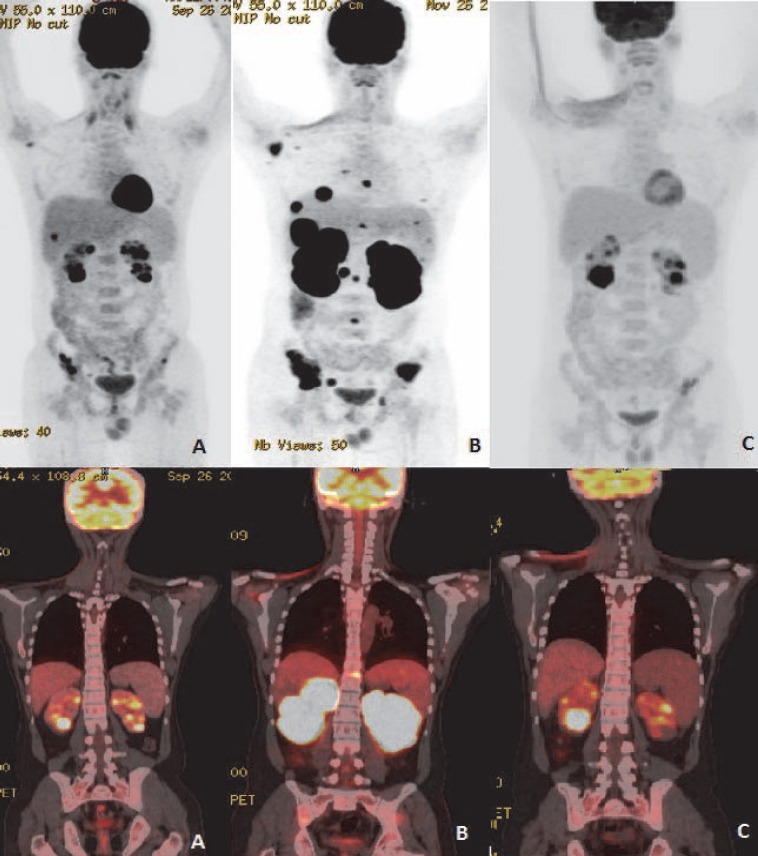
3D MIP and reformatted coronal fused PET/CT images of NHL involving kidney: (A) Multifocal FDG avid cortical lesions involving both kidneys. (B) PET/CT scan for response assessment post 3 cycles chemotherapy shows marked metabolic progression with diffusely increased parenchymal FDG avidity of both kidneys. (C) PET/CT scan done after completion of second line chemotherapy shows partial metabolic response

**Figure 11:**
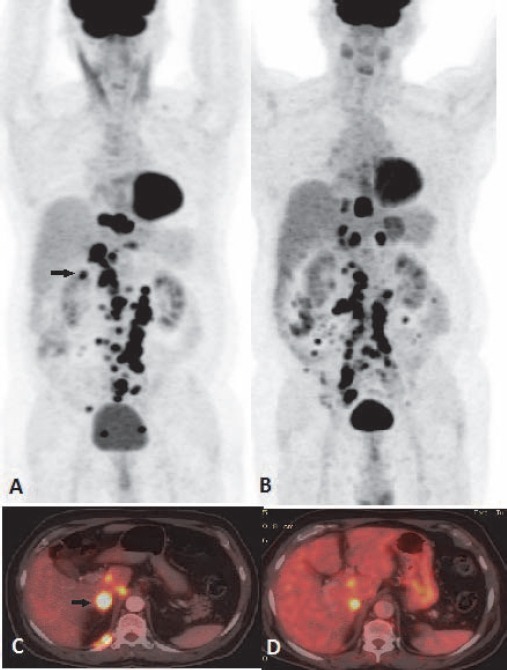
NHL - DLBCL involving adrenal gland: Nodule in right adrenal with increased FDG avidity (A and C). Post therapy scan shows decreased FDG avidity in suprarenal region (B and D)

**Figure 12:**
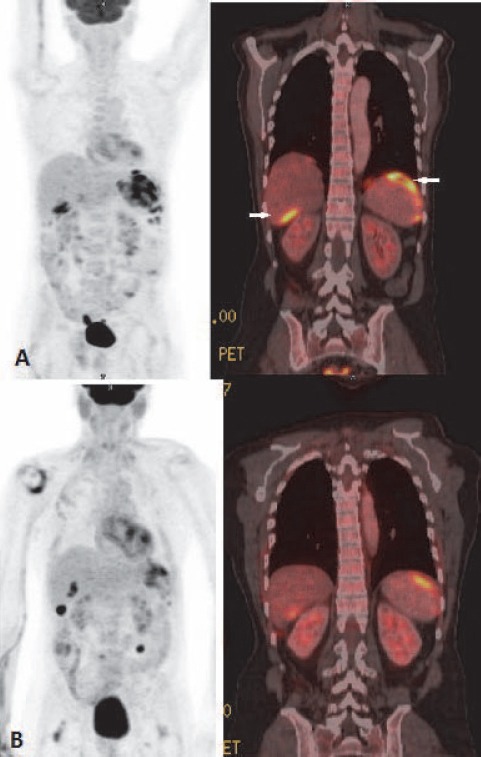
Metabolically active secondary lymphomatous peritoneal deposits in a case of NHL: MIP images and fused coronal images in post 3 cycle response assessment scan (A) show metabolically active plaque like deposit along the parietal peritoneum (arrow). Post treatment scan in lower panel (B) shows partial metabolic response

Other intraabdominal solid organs involved were pancreas (2.1%), kidney (2.1%) and adrenal (1.0%) (Figure 10, 11). Omentum and peritoneum was involved in 2.1 % cases of all extranodal NHL patients (Figure 12).

**Figure 13:**
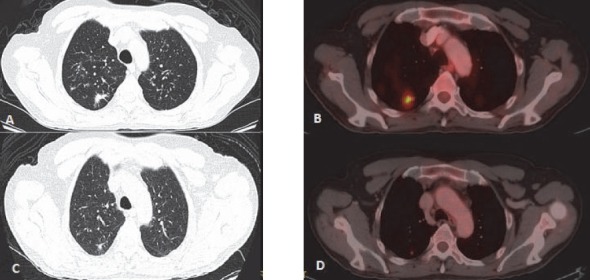
Lymphoma involving lung – Nodular lesion. Primary NHL of small intestine (not in picture). (A) Staging PET-CT scan shows soft tissue parenchymal nodule with irregular margin in right lung. (B) The lesion appears FDG avid. (C) Post 3 cycle chemotherapy shows anatomical reduction of the size of the lesion. (D) Complete metabolic response is seen in post therapy scan

**Figure 14:**
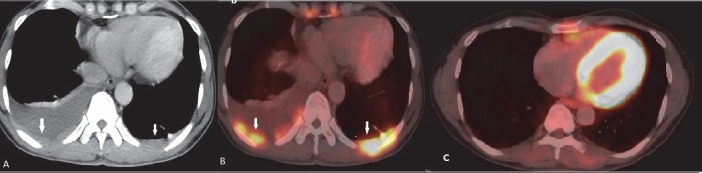
Lymphoma involving the pleura. CT scan shows enhancing pleural nodules (arrows in A) and fused PET/CT image shows increased FDG uptake (arrows in B). Complete metabolic response of the pleural lesions is seen in post chemotherapy scan. Pleural effusion has also subsided in follow up scan (C)

**Figure 15:**
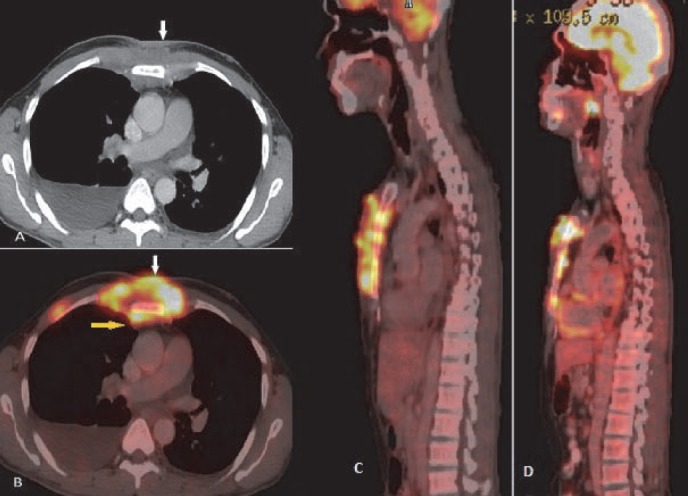
Lymphoma involving the chest wall. CT scan (A) and fused PET/CT images (B) show pre and parasternal ulceroproliferative plaque like lesion (arrow in A) with increased FDG uptake (white arrow in B). Deep extension of the lesion in retrosternal region at the middle 1/3^rd^ of the body of sternum is seen (yellow arrow in B). Post therapy scan shows (D) partial metabolic response as compared to pre therapy scan (C)

**Figure 16:**
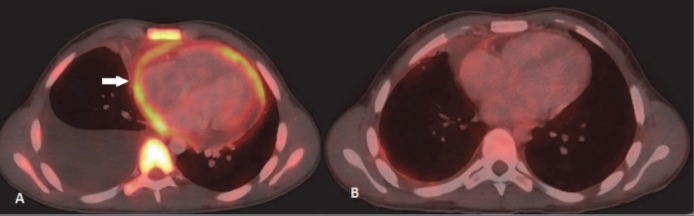
NHL with pericardial involvement. (A) FDG avid pericardial involvement is seen. Associated marrow involvement and pleural effusion is also seen. (B) Post 3 cycle chemotherapy shows complete metabolic response

Histologically proved lung involvement is seen in 3.1% (Figure 13) pleural involvement in 3.1 % (Figure 14) and chest wall involvement in 2.1 % of all extranodal NHL cases (Figure 15). Pericardial involvement with pericardial effusion was seen in one case (Figure 16).

**Figure 17:**
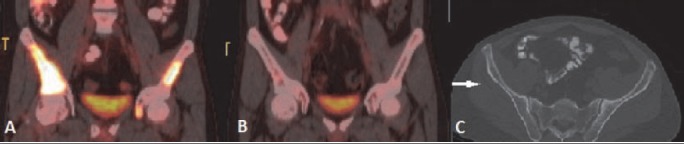
NHL involving bone: (A) PET/CT image shows FDG avid deposit in right ilium. (B) Post chemotherapy complete metabolic response is seen in repeat PET/CT scan. (C) CT scan shows no significant change except minimal cortical irregularity (arrow)

**Figure 18:**
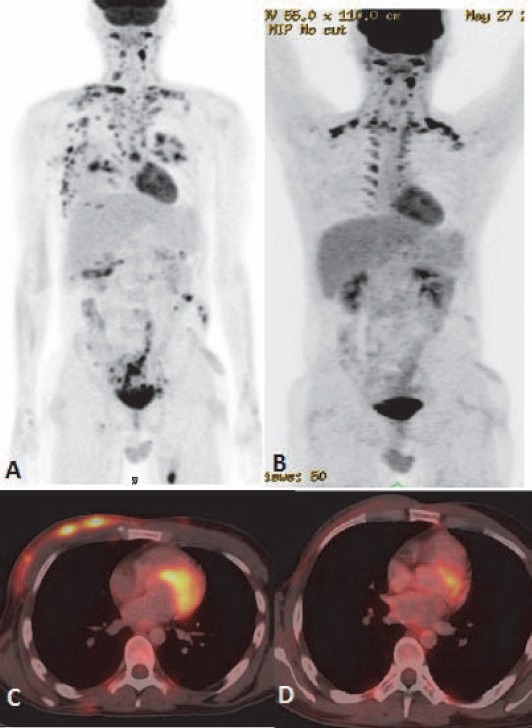
Cutaneous T cell lymphoma: (A) MIP image showing widespread focal radio tracer uptake in chest wall. (C) Corresponding fused PET/CT images shows FDG avid subcutaneous deposits in anterior chest wall. (D)Post therapy scan shows complete disappearance of the lesions. (B) MIP image of post therapy scan shows FDG uptake by brown fat

Bone was involved in 7.4% (Figure 17) and Skin and soft tissue was involved in 2.1% and 1.0% of extranodal cases of NHL respectively (Figure 18).

**Figure 19:**
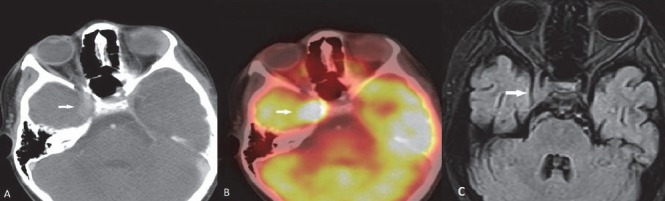
Burkitt’s lymphoma involving CNS: Right cavernous sinus shows enhancing deposit (A) with FDG avidity (B). T2W axial scan of MRI brain through same plane shows soft tissue deposit isointense to gray matter in the region of right cavernous sinus

**Figure 20:**
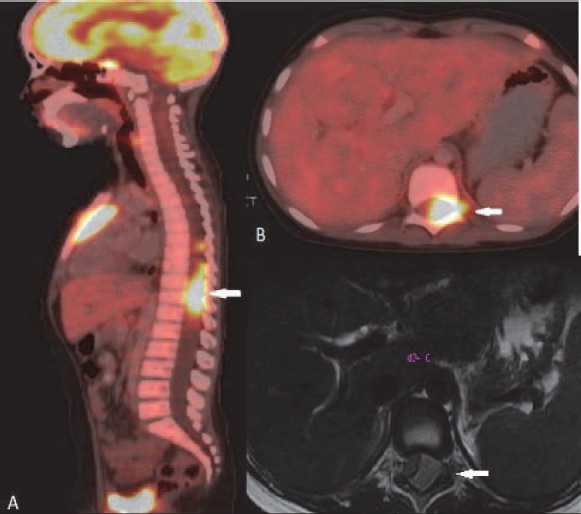
Same patient as in Figure 19. (A) Sagittal fused PET/CT image shows FDG avid intraspinal deposit. Axial fused PET/CT images (B) and axial T2W MRI image (C) confirm the epidural location of the lesion

Primary CNS lymphoma was seen in 2 cases (2.1%). Multifocal involvement of brain and dorsal spine was seen in one patient with primary nodal Burkitt’s lymphoma (Figure 19, 20).

### Hodgkin disease (HD)

Bone was the commonest site of extranodal involvement involving 8 of 26 patients (30.76%). Liver was involved in 11.5% and spleen was involved in 19.2% of patients.

Lung involvement was seen in 19.2% of patients. 3 patients had pleural involvement, among them 2 had involvement of chest wall too.

In our study, skeletal involvement was seen in 15 patients (12.5%). Among them, 3 patients had primary extranodal disease.

Commonest histological subtype of skeletal lymphoma in our study was DLBCL consisting of 36.3% of all skeletal lymphoma.

Axial skeleton is more commonly involved than appendicular skeleton. Dorsal spine is the commonest site in spine involving 36.3% of skeletal lymphoma cases. Among the appendicular skeleton humerus is most commonly involved (18.1% cases).

Routine histopathological examination was not performed in each and every case of extranodal involvement. However, follow up studies of treatment response evaluation and the interpretation of the anatomical and functional images in correlation with the baseline study substantiated that those were indeed part of the same disease process.

## Discussion

### Lymphoma of head and neck

**Figure 21:**
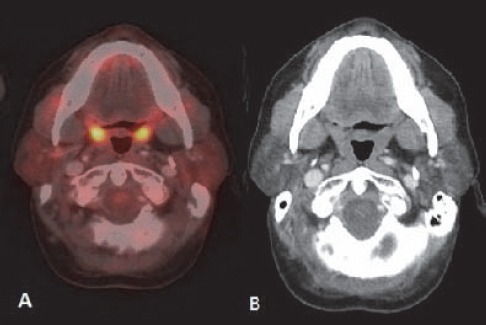
Physiological distribution of radiotracer in bilateral tonsil. No obvious abnormality in corresponding CT scan images (B). Compare with Figure 1. Corresponding change is seen in CT scan in case of pathological radio tracer uptake

After GI tract, the most commonly involved extranodal site for NHL is head and neck region (1). Diffuse large B cell lymphoma (DLBCL) is the commonest histological type (9, 10). The waldeyer ring (including tonsil) is the primary site of 1/3 rd of NHL cases involving head and neck (2). The other organs usually affected are mandible, hard palate, nasopharynx, parotid gland, paranasal sinuses, thyroid gland and orbit. **Tonsil** and other lymphoid tissues of Waldayer’s ring commonly show some amount of physiological or reactive FDG uptake. Symmetrical uptake without any corresponding abnormality in CT scan usually suggests physiological uptake (Figure 21). CT scan increases the specificity of the interpretation of physiological uptake. Asymmetric FDG uptake of unilateral tonsil and nodular FDG avidity with or without enlarged size is helpful in diagnosis of tonsillar involvement in NHL (Figure 1). PET-CT scan as well as MRI may well depict invasion or displacement of adjacent structures. PET-CT scan is particularly helpful in detecting lymphomatous involvement of tonsillar gland of normal size (due to its functional imaging property). In these situations MRI may not be able to detect the lesion (11). **Posterior tongue** is included in the FDG avid part of Waldeyer’s ring. Lymhomatous involvement of posterior tongue appears as a plaque like or ulcerative lesion (Figure 3). Anterior tongue involvement is rare. Lymphoma of **nasopharynx** usually presents with adjacent cervical lymphadenopathy. Increased radiotracer uptake in nasopharynx may be related to physiological radiotracer uptake of lymphoid tissue as well as upper respiratory tract infection. Lymphoma involving the nasopharynx may extend to airways and tonsil (Figure 2). Unlike carcinoma, cranial extension toward skull base is less common (12). **Primary nasal cavity and paranasal sinus** lymphoma are highly aggressive type and demonstrate frequent distant relapse (13). They are hypermetabolic tumours, and ^18^F-FDG PET-CT scan may be useful for detection of local as well as distant nodal and extranodal disease. Regional nodal involvement in cervical nodes is a common finding in PET-CT in head and neck extranodal lymphoma (12). Primary lymphoma of **thyroid gland** is rare. Thyroid lymphoma is typically NHL and B-cell type (1). Thyroid gland also may show physiological radiotracer uptake. Diffuse thyroid uptake may be seen in post chemotherapy state. PET-CT features of thyroid lymphoma are not specific. Focal FDG uptake is more commonly associated with primary thyroid malignancy. Diffusely increased FDG accumulation may be associated with thyroiditis (eg. Hasimoto’s thyroiditis) (14). Low level diffuse FDG avidity is also seen in post chemotherapy reactive thyroiditis. Primary NHL of **salivary gland** is relatively uncommon. Parotid gland is most commonly affected. Most common subtype is MALT. It is commonly associated with Sjogren syndrome (1). Lymphoepithelial sialadenitis (LESA) or Myoepithelial sialadenitis (MESA) are morphological conditions of salivary glands which predispose to salivary gland lymphoma. LESA/MESA can give rise to lymphoma of parotid gland with or without clinical manifestation of Sjogren syndrome (15). Secondary involvement of parotid gland is more commonly seen in DLBCL (Figure 5). PET-CT scan interpretation of **ocular disease** in lymphoma may be diffcult due to combined effect of small volume of lesion and close proximity of lesion to the brain where physiological radiotracer uptake is high. (2). Ocular adnexal lymphoma refers to lymphoma in extraoccular orbital space involving lacrimal gland, orbital soft tissue, conjunctiva and eyelids. Orbial lymphoma usually presents as soft tissue mass arising from conjunctiva or other elements of orbit (Figure 4). Extraoccular muscle may be surrounded or displaced by the lesion. Globe or optic nerves are usually not affected.

### Lymphoma of thorax

Intrathoracic involvement is more common in HD than in NHL (1). ENL may involve lung, pleura, myocardium, pericardium, thymus, chest wall or breast. Lung or chest wall may be involved directly from mediastinal nodal disease or by haematogenous spread. Direct involvement from the nodal disease indicates better prognosis. Lung parenchyma is one of the common sites for disease recurrence in lymphoma. On CT scan, **pulmonary parenchymal** involvement shows variable characteristics. The most common pattern is direct extension from nodal disease. Other presentations include parenchymal nodule (Figure 13), rounded or segmental consolidation with or without airbronchogram, peribronchial nodular or linear septal thickening. None of the parenchymal disease pattern is characteristic of lymphoma. In staging CT, differential diagnosis of FDG avid lung lesion includes benign condition like granulomatous disease or a second primary malignancy. In post therapy follow up study possibility also include pneumonitis or chemotherapy induced changes (16). HD of the lung parenchyma is relatively rare and is usually due to direct extension from mediastinal disease (1). Reactive **pleural effusion** is common and is usually accompanied by mediastinal lymphadenopathy. They are usually exudates secondary to central lymphatic or venous obstruction. Focal FDG uptake of pleura associated with effusion help in differentiation of active pleural disease from reactive effusion. Focal pleural lesion can be seen as plaques, discrete nodules or a bulky mass lesion (Figure 14). Focal pleural masses are more commonly seen in recurrence (17). Increased FDG uptake of **pericardium** along with pericardial effusion is indicative of lymphomatous involvement of pericardium (Figure 16). Reactive pericardial effusion associated with large mediastinal mass adjacent to heart is easily seen in CT. For staging, effusion is considered as evidence of pericardial involvement (17). Pericardial lymphoma has also been described in NHL (18). Direct spread of lymphoma to heart is more common in patient with AIDS related lymphoma (ARL) and post treatment lymphoproliferative disorder (PTLD). Retrograde lymphatic spread or haematogenous spread to heart is rare. Physiological FDG uptake in the myocardium may mask the lymphomatous involvement. Hodgkin’s disease involving the heart and pericardium is very rare (19). Primary **oesophageal** lymphoma is rare. Most cases are direct extension from involved mediastinal nodes. Most common subtype is DLBCL. At PET/CT scan, circumferential thickening with diffuse FDG uptake is seen. Biopsy may be needed to differentiate it from carcinoma. Primary involvement of **thymus** in HD is rare. However, involvement of the thymus does not alter the disease staging, since the thymus is considered to be a nodal organ (1). Mediastinal large B cell lymphoma involves the thymus. Young women are commonly affected. The disease grows rapidly and may obstruct the superior vena cava (SVC). On CT scan, differentiation of enlarged thymus from mediastinal lymph node enlargement is often difficult as both have homogenous soft tissue density or heterogenous nodular appearance. However, thymic disease usually retains the shape of the gland whereas the nodal disease is usually lobulated. Occasionally, cystic areas are demonstrated in thymic lesion which may even increase in size following regression of rest of the gland after treatment. Calcification may present at onset or may develop during treatment. Benign rebound thymic hyperplasia may develop often following completion of chemotherapy and usually presents as a diffusely FDG avid anterior mediastinal lesion. The intensity of the uptake is usually mild to moderate. The condition may be mistaken for disease recurrence. High FDG avidity of thymus may decrease or disappear at repeat imaging several weeks later. Absence of active disease elsewhere in body is usually associated with rebound thymic hyperplasia and that fact may help in diagnosis. Biopsy may be required at times. The most common presentation of **chest wall** is direct invasion from anterior mediastinal mass (Figure 15). Nodal disease from internal mammary chain is the most common offender, but it can also spread from axillary or supraclavicular node. More aggressive treatment is indicated for chest wall disease because of higher recurrence rate (20). Primary lymphoma of **breast** is rare. Lymphoma of breast is usually a part of widespread disease elsewhere. Most common type of unilateral breast lymphoma is DLBCL. Burkitt’s lymphoma is less common and presents as bilateral diffuse disease. The age distribution of lymphoma of breast is bimodal. First peak occurs during pregnancy and lactation and is often high grade. The second peak is around 50 years and it is most commonly a solitary lesion. The secondary involvement is usually characterized by nodules with associated large volume of lymphadenopathy. As mammography is not routinely performed in lymphoma staging and CT is not accurate for breast pathology, the whole body imaging capability of PET enable identification of unexpected involvement of the breast (2). PET/CT scan may be very helpful in dense breast, where lymphomatous involvement of breast tissue is characterized by increased FDG uptake.

### Lymphoma of Abdomen

**Figure 22:**
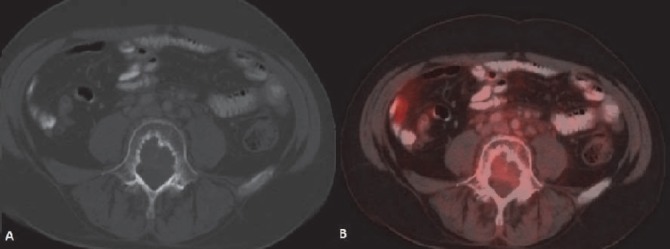
Post radiotherapy bone lesion of lymphoma showing metabolically inert lytic lesion of L4 vertebra with marginal sclerosis

The gastrointestinal tract (GIT) is most commonly involved in NHL. Around 10% of all NHL cases show lymphomatous involvement of one or other sites of GIT (6). Commonly involved organs are stomach, small bowel, large bowel and oesophagus. Usually, DLBCL and MALT lymphoma are common histological types involving gastrointestinal organs (21). Enteropathy associated T-cell lymphoma is common among patients with Coeliac disease (6). HD rarely involves GIT and it is usually involved from adjacent mesenteric or retroperitoneal lymph nodes. When GIT is involved, usually a single site is affected and it indicates poor prognosis (22). **Stomach** is the most common (around 50 %) site for GIT lymphoma (1). Secondary gastric involvement is more common than primary lesion. Primary disease is common in NHL. Low grade MALT lymphoma and high grade DLBCL are the most common histological subtypes. Association with Helicobactor pylori is well known. Three patterns are usually seen – polypoid, ulcerating and infiltrative. Radiological features may be indistinguishable from gastric carcinoma. Features suggestive of gastric lymphoma include – multiple polypoid tumours, giant cavitating lesions, and extensive infiltration with pronounced thickening of gastric fold without predilection for any one site (Figure 6). Diffuse submucosal infiltration simulating linitis plastica is also seen. Less desmoplastic reaction with preserved distensibility differentiates it from carcinoma. Transpyloric extension is more common in lymphoma. Unlike gastric carcinoma contiguous infiltration to adjacent organ is rare. Normal physiological uptake may pose a problem. At PET/CT the uptake of the lesions are higher than the liver uptake in majority of the cases. (23). Adequate distension of the stomach with oral contrast is important. PET-CT is particularly useful in detecting extragastric involvement. **Small bowel** is second most commonly affected organ in GIT (after stomach). Distal ileum is most frequently affected and incidence of involvement appears progressively less frequent proximally. Iliocaecal region is particularly affected in children. Disease is multifocal in 50 percent cases. Mantle cell lymphoma is most frequent histological subtype. (24). Focal or diffuse bowel wall thickening with alternating areas of dilatation (due to nerve plexus destruction) or constriction of lumen are seen. Folds in affected segment appear thick, nodular or effaced. Primary **colonic** lymphoma is Burkitt or MALT subtype. Caecum and rectum are most common sites (Figure 7). Diffuse or segmental distribution of small nodules are the most common pattern in PET-CT. Solitary polypoid mass is less common, which is seen in caecum and indistinguishable from carcinoma. Concomitant involvement of terminal ileum is more suggestive of lymphoma. Lymphomatous involvement of rectosigmoid causes larger segment of stricture than adenocarcinoma and irregular excavation of mass (17). Secondary **hepatic** involvement in lymphoma is more common than primary disease. As hepatic metastasis from solid tumour, focal hepatic deposit is common in liver. However, lymphomatous deposits in liver are usually smaller in size and may not be usually associated with splenic lesion. In PET-CT scan patchy uptake of radiotracer of the focal lesions are seen with SUV greater than adjacent normal parenchyma. Associated increased uptake at portal and retroperitoneal nodes are common findings in secondary lymphomatous involvement of liver. The **spleen** is considered extranodal region in NHL and nodal organ in HD (1). Splenic involvement is more common in HD. Organ size is not a criterion for diagnosis as spleen can be normal in size with tumor infiltration or may be enlarged without lymphomatous involvement (2). ^18^F-FDG PET-CT has 100 percent sensitivity and specificity in diagnosing primary splenic involvement (25). In post therapy scan the sensitivity decreases because of reactive splenic uptake. Diffuse increased FDG activity greater than liver (Figure 9) and intense focal uptake with or without corresponding CT lesions (Figure 8) are the usual patterns helpful to diagnose splenic involvement. Diffuse **peritoneal** lymphomatosis is often associated with high grade gastrointestinal NHL. It manifests as discrete nodules or large infiltrative mass (Figure 22). Ascites may be present. ^18^F-FDG PET may show mild diffuse FDG concentration of ascites with high activity of the peritoneal nodules or mass. Large irregular soft tissue mass in mesentry attached with bowel loops are frequent finding in lymphoma. Majority of cases of **pancreatic** lymphoma are secondary to contiguous lymph node disease as the pancreas has no capsule. The characteristic PET/CT imaging feature is focal or diffuse radiotracer uptake in the pancreatic tissue. Primary involvement of pancreas in NHL is rare (26). **Kidney** is usually involved in aggressive form of NHL. Because FDG is normally excreted by kidney, PET imaging is not sensitive for renal lymphoma. Fused images show single or multiple cortical focal areas of FDG uptake with or without corresponding CT lesions. Less commonly solitary mass in one pole of kidney or oedematous enlargement of the whole organ with diffuse FDG avidity is seen (Figure 10). Primary **adrenal** lymphoma is very rare. Secondary involvement of adrenal is common in NHL. ^18^F-FDG PET/CT is useful in differentiating adrenal involvement from incidentaloma (Figure 11). CT features are also important to increase the specificity. Primary lymphoma of **genital tract** is rare. Of the female genital organs, adnexa is most common site secondarily involved in NHL (1). DLBCL is the commonest subtype. Involvement of the body of the uterus and cervix is also noticed. Involvement of vulva and vagina are rare. PET/CT shows foci of abnormally increased uptake in adnexa and uterus. Physiological uptake in ovaries and endometrium in premenopausal age may lead to false positive result. Primary **testicular** lymphoma is rare and a highly aggressive disease. Secondary involvement of testis is seen usually in extensive disease. Testicular lymphoma appears as FDG avid focal lesion. Background physiological uptake of FDG in testes is variable and may cause problem in diagnosis.

### CNS lymphoma

Intense physiological uptake of ^18^F-FDG in brain parenchyma hinders intracranial lymphomatous involvement. Steroid therapy given for management for intracranial pathology can also lead to false negative FDG PET/CT study (2). Mainstay of primary diagnosis and therapy monitoring of primary CNS lymphoma is MRI. PET/CT may help identifying recurrence or exclude disease (Figure 19, 20). Delayed and dedicated brain PET in selected cases will be helpful.

### Lymphoma of the Bone

When a lymphomatous lesion is originating from bone it is considered as stage I disease, but bone involvement associated with disease originating from other than bone indicates stage IV disease (1). Most common primary as well as secondary skeletal lymphoma is DLBCL. Primary osseous NHL usually arises from appendicular skeleton or from the flat bones of the axial skeleton. Secondary osseous disease commonly involves the axial skeleton. Imaging features of osseous lymphoma are non specific and usually suggestive of aggressive disease (Figure 17). ^18^F-FDG PET is more sensitive and specific than bone scintigraphy (27).

Identification of bone marrow involvement is essential before treatment of NHL as well as HD. Bone marrow biopsy is considered the gold standard. ^18^F-FDG PET is highly sensitive in detection of bone marrow disease (28, 29). Two patterns of bone marrow involvement are seen –diffuse or focal mono or polyostotic bone marrow disease. PET/CT can also be used for guided biopsy. Bone involvement may be predominantly osteolytic, mixed lytic and sclerotic or predominantly sclerotic. Sclerotic lesion may arise de novo or following radiotherapy to a lytic lesion (Figure 22). Moth eaten appearance in diaphysis, characteristic of round cell tumour is also seen.

**Figure 23:**
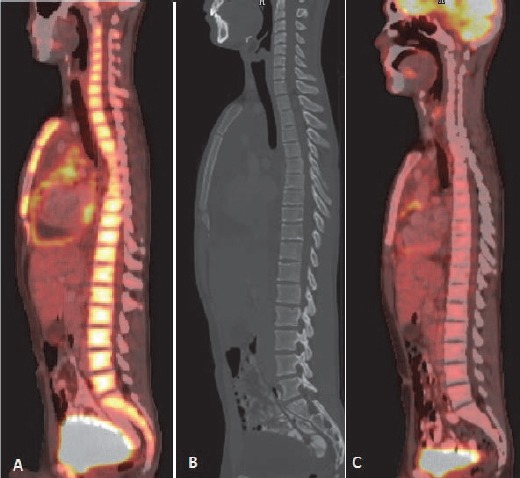
Diffusely increased radio tracer uptake seen in visualised axial skeleton post chemotherapy suggestive of marrow hyperactivation (A). CT scan shows no cortical abnormality (B). Follow up scan shows normal marrow uptake of radio tracer (C).

After treatment, mainly chemotherapy and granulocyte colony stimulating factor (G-CSF) diffuse marrow uptake of radiotracer is often seen (Figure 23). Increased splenic uptake is commonly associated (23).

### Cuteneous Lymphoma

Cutaneous lymphoma may be primary cutaneous lymphoma or secondary to disseminated disease. Primary cutaneous lymphoma is common in NHL. It has various subtype depending on clinical behaviour, prognosis and FDG avidity. 65% of primary cutaneous lymphoma is T-cell lymphoma, reminder being B-cell lymphoma (1). Primary and secondary skin involvement is very rare in HD. Extracutaneous manifestation is important in determining prognosis of disease and treatment planning. ^18^F-FDG PET/CT provides adequate metabolic and anatomic information (Figure 18).

### Muscular Lymphoma

Muscle is a very uncommon site for primary extranodal involvement and indicates poor prognosis (31). Hematogenous dissemination of disease from other site is common. PET/CT shows single or multiple intramuscular foci and helps in guided biopsy.

## Conclusion

In our study DLBCL was the commonest histological subtype in extra nodal involvement and GI tract was the commonest site of involvement in NHL

The commonest site of extranodal involvement in HD was the skeletal system. DLBCL was found to be the most common histological subtype with maximum number of skeletal involvement in the published literature. In our study population nearly equal number of HD and DLBCL patients showed signs of skeletal involvement.

GI tract was the commonest site of extra nodal involvement in NHL. Interestingly no HD patient showed GI tract involvement. Eleven cases of Waldeyer’s ring and tonsillar involvement were noticed in the NHL group but only one HD patient shows tonsillar involvement.

Prevalence of extranodal lymphoma is increasing. This article illustrates the various PET/CT appearances of usual and unusual forms of extranodal involvement in lymphoma.
